# Analysis of Saccade Characteristics During Fusional Vergence Tests in Normal Binocular Vision Participants

**DOI:** 10.3390/jemr19010015

**Published:** 2026-02-03

**Authors:** Cristina Rovira-Gay, Clara Mestre, Marc Argilés, Jaume Pujol

**Affiliations:** Centre for Sensors, Instruments, and Systems Development (CD6), Universitat Politècnica de Catalunya-BarcelonaTech (UPC), 08222 Barcelona, Spain; clara.mestre@upc.edu (C.M.); marc.argiles@upc.edu (M.A.); jaume.pujol@upc.edu (J.P.)

**Keywords:** binocular vision, fusional vergence, eye tracking, saccades detection

## Abstract

The purpose of the study was to analyze, characterize, and compare the measurements of saccades that occurred during the positive and negative fusional vergence test (PFV and NFV, respectively) as a function of the disparity vergence demand. Thirty-four participants’ PFV and NFV amplitudes were measured in a haploscopic setup, recording eye movements with an Eyelink 1000 Plus (SR Research). The visual stimulus was a column of letters. Break and recovery points were determined objectively offline, and saccades were detected with a velocity-threshold-based method. A total of 13,103 and 14,381 saccades were detected during the measurement of the PFV and NFV ranges, respectively. Saccades followed the main sequence (ρ = 0.97, *p* < 0.001). The distributions of saccadic amplitudes during PFV and NFV differed significantly (U = 4.28, *p* < 0.001). The amplitude of saccades that occurred while fusion was maintained (median (IQR) 0.73 (0.92) deg) was significantly smaller than that of saccades during diplopia (2.10 (3.90) deg) (U = −75.63, *p* < 0.001). The distributions of saccade direction during the measurement of PFV and NFV amplitudes were statistically significantly different (*p* < 0.01). These findings contribute to a better understanding of how the visual system adjusts saccades in response to different disparity vergence demand during fusional vergence amplitudes evaluation.

## 1. Introduction

The fusional vergence test is a crucial diagnostic tool in optometric clinical practice for assessing binocular function and diagnosing binocular vision dysfunctions [1]. Abnormally low fusional vergence amplitudes may lead to symptoms such as visual tiredness, headache, or double vision, particularly during sustained near work [2]. The fusional vergence test involves assessing disparity-driven convergence and divergence movements near or at distance, and is commonly performed in clinics using two tests: the step vergence test, conducted using a prim bar to induce disparity; and the smooth vergence test, conducted using rotatory Risley prisms [3,4].

Base-in prisms are used to elicit divergence and, therefore, measure negative fusional vergence (NFV) amplitudes, whereas base-out prisms are employed to elicit convergence and measure positive fusional vergence (PFV) amplitudes. The test consists of gradually increasing the vergence demand until fusion is disrupted (when patients report double vision), identifying the “break point”. Then, the vergence demand is reduced until fusion is restored and the patient reports single vision. The prism power at this point is the “recovery point” [1,5]. Hence, vergence break and recovery points are critical events in the fusional vergence test, indicating the limits of an individual’s ability to maintain binocular fixation [1,5,6].

There are substantial differences between convergence and divergence movements. Convergence generally yields larger amplitudes than divergence in healthy subjects because the physiological convergence mechanism tends to have higher reserves [7]. Also, the neural pathway of convergence is better documented compared to divergence, as, for divergence, there is less clear evidence of distinct neuronal populations; some divergence movement may be mediated by a reduction/inhibition of convergence neurons rather than a dedicated strong divergence pathway [8]. Clinically, low PFV amplitudes may suggest convergence insufficiency; low NFV may suggest divergence insufficiency or inability to relax convergence [8]. Vision therapy [9] has been found to be more effective to train or improve convergence than divergence [10]; while it is the treatment of choice in cases of convergence insufficiency [11], its effectiveness in cases of divergence insufficiency is less clear [12].

The subjectivity of the fusional vergence test, which relies on the patients’ responses and/or examiner criteria, contributes to low intra-examiner and inter-examiner reliability, and results are variable [13,14,15,16]. This limitation can be overcome by measuring fusional vergence amplitudes objectively using eye tracking systems. Additionally, objective recordings of vergence eye movements offer a more detailed characterization of eye movement physiology compared with traditional optometric clinical testing methods [17,18,19]. For example, involuntary eye movements that occur during vergence tests, such as saccades and microsaccades, cannot be characterized with conventional methods [20,21]. Saccadic eye movements are rapid ocular movements that are present during the smooth vergence test [22].

Saccades can be generated as a consequence of an involuntary reflex to fixate on a stimulus appearing suddenly on the visual field, or voluntary movements to be executed deliberately to alter gaze. Microsaccades, on the other hand, are miniature, involuntary eye movements occurring during fixation [23]. Involuntary reflex saccades can be considered as larger microsaccades (larger than 1 deg) [24], so both eye movements are present during fixation [25].

Eye tracking technology can be used to measure and analyze the eye movements of participants undergoing vergence tests [18,21,26]. Objective measurement of fusional vergence amplitudes has been undertaken in prior research [10,18,19,26,27,28]. Likewise, saccadic and vergence behavior has been studied to demonstrate the improvement of fusional vergence amplitudes after vision therapy protocols objectively in patients with convergence insufficiency [19,27,29,30,31], and with concussion-related convergence insufficiency patients [28]. In addition, vergence symmetry [32], the effect of repetition on vergence [33], and the relationship between vergence latency and age [34] have been well studied.

Several algorithms have been proposed to detect saccades and microsaccades from eye tracking recordings. Velocity-threshold-based algorithms, such as the one proposed by Engbert and Kliegl [35] and modified subsequently by Engbert and Mergenthaler [36], have been used extensively to identify saccades. This algorithm relies on the fact that the mean horizontal and vertical velocities during fixation are zero and detect saccades based on an adaptive velocity threshold [35]. Nystrom and Holmqvist developed another adaptive velocity-threshold algorithm to detect not only saccades, but also fixation and glissades [37]. Other approaches can be also used, such as the saccade detector based on an unsupervised clustering method developed by Otero-Millan et al. [38].

The goal of this study was to analyze the characteristics of saccades that occur during the positive and negative fusional vergence test. This study analyzed measurements such as amplitude, peak velocity–amplitude relationship (main sequence), direction, and frequency of saccades occurring during the fusional vergence test as a function of the disparity vergence demand. By examining these behaviors, this study aims to understand how the oculomotor system responds in demanding vergence situations.

## 2. Materials and Methods

### 2.1. Participants

A total of 34 young adults with a mean ± standard deviation (SD) age of 23.24 ± 2.39 years, including 9 males and 25 females, predominantly university students, were included in this study. The study’s inclusion criteria encompassed individuals aged 19 to 29 years, corrected monocular visual acuity of 0.05 LogMAR or better at both distance and near vision with spherical refractive errors ranging from +3.00 D to −3.00 D, astigmatism below 1.50 D, anisometropia less than 1.00 D, no history of strabismus, and no use of active orthokeratology treatment. All participants with a prescription were wearing soft contact lenses during the test. In addition, to ensure normal binocular vision, participants could have exhibited a maximum of two of the following clinical signs: exophoria of 10 prism diopters (Δ) or greater at near, esophoria of 2 Δ or greater at near, near point of convergence break point exceeding 6 cm, monocular amplitude of accommodation lower than values predicted by Hofstetter’s equation (18.5 − 0.25 × age), monocular and binocular accommodative facility lower than seven cycles per minute (cpm), and vergence facility lower than 9 cpm [39]. In addition, participants demonstrated a score below 16 in the Convergence Insufficiency Symptom Survey (CISS) questionnaire [40].

All participants were informed about the nature of the study, including procedures and aims, as well as their rights and the possibility to withdraw at any time without consequences. All participants signed a written informed consent form prior to their participation. The study followed the tenets of the Declaration of Helsinki and was approved by the Ethics Committee of Hospital Mutua de Terrassa (Terrassa, Spain), identification number: P/21-093.

### 2.2. Equipment and Procedure

**Figure 1:**
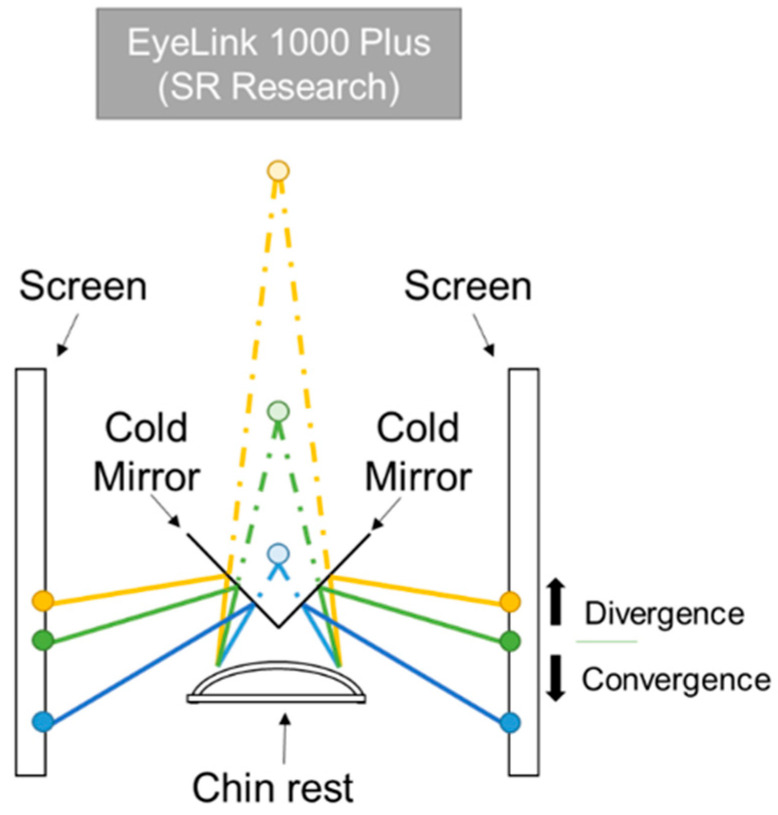
Schematic representation of the haploscopic system and representative examples of vergence movements that could be elicited in the experimental setup. The three circles on each screen show different stimulus positions. The two green circles induce a disparity equivalent to a stimulus placed at 40 cm in front of the subject, shown by the light green circle, and it represents the starting point of the test. Compared to this, when the stimuli on each screen move in the direction of the yellow circles, fusional divergence is stimulated, whereas when the stimuli move towards the blue circles, fusional convergence is elicited.

The fusional vergence test was implemented in a haploscopic system (Figure 1) using a column of 0.20 LogMAR letters as fixation stimulus. A haploscope is an optical setup that allows dichoptic presentation (presentation of distinct stimuli to each eye simultaneously). The haploscopic setup used in the study consisted of two equal monitors with a resolution of 1920 × 1080 pixels (angular pixel size of 2.37 arc min at 40 cm) and frame rate of 60 Hz [18]. Each eye could see the stimulus presented in one of the monitors thanks to two cold mirrors, which transmitted infrared wavelengths, oriented at an angle of 45 deg relative to each monitor. An EyeLink 1000 Plus (SR Research Ltd., Kanata, ON, Canada) was used to measure eye movements at a sampling rate of 500 Hz, and was placed at 50 cm in front of the participant. As it operates in the infrared range, it could track the eyes through the cold mirrors.

The stimuli presentation on each monitor was controlled with custom software developed in Matlab R2020b (MathWorks, Inc., Natick, MA, USA) and the Psychophysics Toolbox [41,42,43]. Prior to obtaining objective measurements of fusional vergence amplitudes, a monocular centering procedure was done to establish the position on each screen that corresponded to a baseline convergence at 40 cm. This position differed among participants based on their individual interpupillary distance. Subsequently, monocular calibration and validation procedures were performed using the EyeLink’s built-in 9-point calibration. The experimental tests started immediately after the calibration procedures were completed.

Fusional convergence and divergence movements were driven by changing the stimuli position synchronously in the two screens from the baseline position (0 prism dioptres, Δ). The stimuli moved smoothly at 1 Δ/s to increase the vergence demand up to 45 Δ (equivalent to 24.2 deg) of symmetric convergence and divergence to mimic the stimulation of the clinical measure using rotatory Risley prisms. After reaching the maximum vergence demand, stimulus disparity was decreased at the same velocity until the baseline position (0 Δ). Therefore, the measurement of NFV amplitude entailed divergence movements followed by convergence to return to the baseline position, whereas PFV amplitude measure consisted of convergence followed by divergence movements. In order to mitigate the influence of vergence adaptation, NFV amplitude was measured before PFV amplitude [44,45]. Each fusional vergence sign was tested three times, with a brief break in between to minimize the potential for fatigue. Each repetition took 90 s. Before each measurement of fusional vergence amplitudes, the centering, calibration, and validation procedures were repeated.

All experiments took place in a dark room to minimize the impact of proximal vergence. Consequently, the primary visual cue that drove vergence in this study was disparity.

### 2.3. Eye Movements Data Analysis

Eye movements data analysis was performed offline using Matlab R2020b. The fusion break and recovery points were determined offline using a custom algorithm for the analysis of vergence movements [18,26]. First, periods of 200 ms before and after each blink identified by the EyeLink software were removed and filled by linear interpolation. Vergence was computed by subtracting left and right eyes’ horizontal gaze positions. Fusional vergence break points were determined with an iterative least-squares fitting procedure. A straight line was fitted to the vergence position over time iteratively, adding 0.10 s of data in each iteration. Then, the break point corresponded to the vergence demand at the time of the last fit before the coefficient of determination of the fit (R^2^) started to decrease. The same procedure was done to find the recovery point. The mean ± SD R^2^ that determined the break and recovery points was 0.979 ± 0.026 for NFV and 0.982 ± 0.037 for PFV. A break point of 45 Δ was assigned to participants who did not exhibit loss of motor fusion, and no recovery value was recorded in these cases.

The velocity-threshold-based algorithm proposed by Engbert and Kliegl [35] and modified subsequently by Engbert and Mergenthaler [36], with λ = 6 and a minimum duration of 6 ms, was used to identify saccades. A minimum intersaccadic interval of 20 ms was imposed. Therefore, two detected saccades separated by less than 20 ms were fused into a single movement. Although an amplitude threshold of 1° is commonly adopted to distinguish microsaccades from saccades, these events are treated as a continuum in the present study [25].

### 2.4. Statistical Analysis

Matlab and IBM SPSS 27.0 for Windows were used for statistical analysis. Significance was determined at a level of *p* < 0.05. Normality of all variables was evaluated with the Shapiro-Wilk test. Parametric tests were employed for normally distributed variables, and nonparametric tests were used when normality could not be assumed. In addition, circular statistics analyses were performed to analyze the direction data using the Circular Statistics Toolbox for Matlab [46] and R version 4.3.3 using the circular package (version 0.4-3) to test for homogeneity of direction distributions between two groups using the Watson–Wheeler test [47].

## 3. Results

The means and SD of fusional vergence amplitudes were 13.62 ± 4.28 Δ and 10.26 ± 4.50 Δ for NFV amplitude break and recovery points, respectively, and 37.37 ± 9.47 Δ for the break and 27.70 ± 9.11 Δ for the recovery points of PFV amplitude.

The number of detected saccades during the measurement of positive and negative fusional vergence amplitudes was 14,381 and 13,103, respectively. For all detected saccades, 47.65% had amplitudes < 1°, 40.76% were between 1 and 5°, 10.62% between 5 and 15°, and only 0.97% exceeded 15°. Considering the direction of the underlying vergence movement, 59.85% of the saccades done during PFV amplitude measurement were <1 deg, 34.56% were between 1 and 5 deg, 4.85% were between 5 and 15 deg, and 0.74% were >15 deg; whereas a higher proportion of saccades done during NFV amplitude measure tended to show greater amplitude: 34.25% were <1 deg, 47.57% were between 1 and 5 deg, 16.95% were between 5 and 15 deg, and 1.23% were >15 deg.

**Figure 2:**
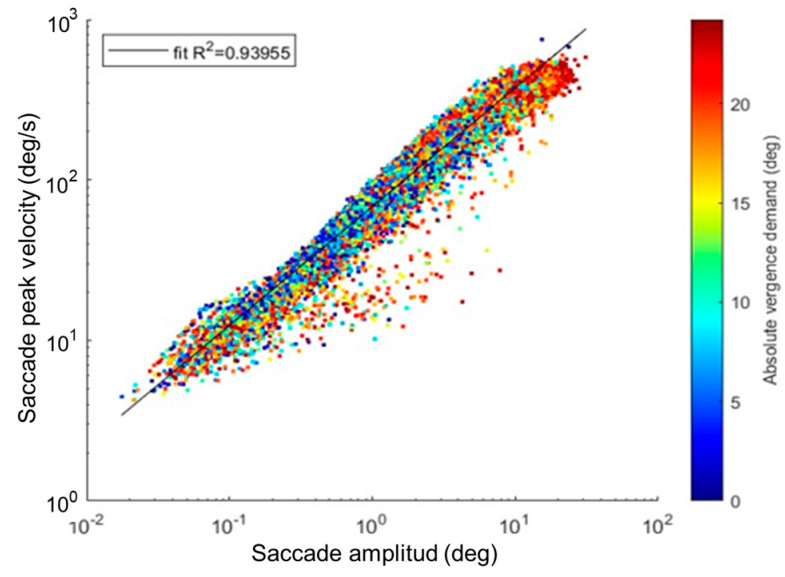
Main sequence for all detected saccades. The color bar represents the absolute vergence demand in degrees. It shows a gradation of colors from blue (indicating low vergence demand) to red (indicating higher vergence demand).

All saccades followed the main sequence, with a strong correlation between amplitude and peak velocity (Spearman’s correlation, ρ = 0.97, *p* < 0.001) (Figure 2). Both the saccades done during PFV amplitude measurement (ρ = 0.96, *p* < 0.001, linear fit R^2^ = 0.931) and the saccades done during NFV amplitude measurement (ρ = 0.97, *p* < 0.001, linear fit R^2^ = 0.938) exhibited comparable statistical significance and robustness in the regression analysis. Similar results were found for saccades that occurred during single vision (ρ = 0.97, *p* < 0.001, linear fit R^2^ = 0.938) and diplopia (ρ = 0.97, *p* < 0.001, linear fit R^2^ = 0.914).

### 3.1. Difference Between the Number of Saccades

The average saccade rate across the three repetitions of positive and negative fusional vergence measures was 1.42 ± 0.37 saccades/second and 1.56 ± 0.43 saccades/second, respectively. Considering the direction of vergence movements, 1.46 ± 0.37 saccades/second were performed during divergence and 1.53 ± 0.38 saccades/second were performed during convergence. Paired t-tests showed statistically significant differences between the two compared pairs, indicating variations in saccade rate between tasks involving divergence and convergence (t(33) = −2.55, *p* = 0.015), and between positive and negative fusional vergence measures (t(33) = −2.53 *p* = 0.016).

Descriptive statistics for the number of saccades performed in each repetition are presented in Table 1. Friedman tests showed no statistically significant differences in the number of saccades performed in the three repetitions of NFV amplitude measure (χ^2^(2) = 0.72, *p* = 0.697). However, statistically significant differences in the number of saccades done during the repetitions of PFV measure were found (χ^2^(2) = 11.30, *p* = 0.004). This difference was greater between repetitions 1 and 3 (z = −2.62, *p* = 0.009) than between repetitions 1 and 2 (z = −1.82, *p* = 0.069) and 2 and 3 (z = −1.93, *p* = 0.054). As the measurement of PFV amplitude advanced, participants made more saccades in each repetition.

### 3.2. Amplitude Distribution

**Figure 3:**
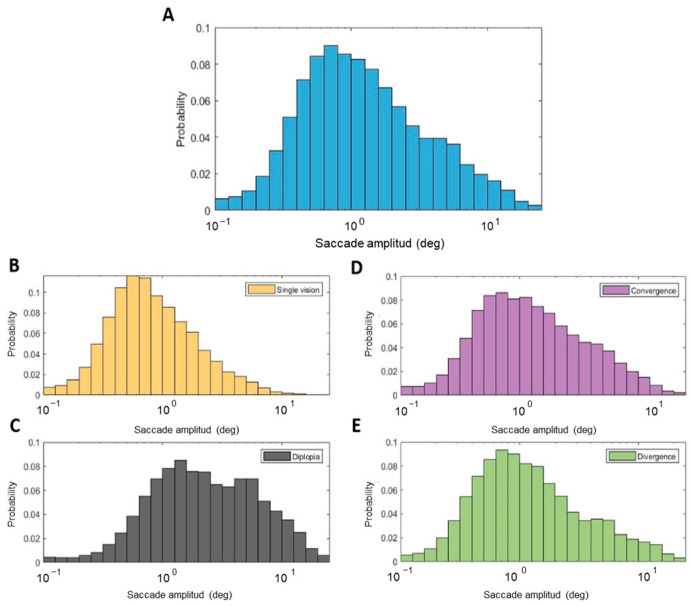
Saccades’ amplitude distributions. (**A**) Saccades’ amplitude distribution for all saccades. (**B**,**C**) represent the amplitude distributions of saccades done during the experience of single vision and diplopia, respectively. (**D**,**E**) represent the amplitude distributions of saccades done during convergence and divergence movements, respectively.

The median (interquartile range; IQR) of all saccades’ amplitude distribution (Figure 3A) was 1.06 deg (1.84 deg). The algorithm used to determine the break and recovery points of fusional vergence amplitudes allowed us to distinguish the periods when participants experienced diplopia and single vision (fusion). During the periods of diplopia, participants tended to exhibit larger saccades (2.10 deg (3.90 deg)) than during periods of single vision (0.73 deg (0.92 deg)) (Figure 3B,C). This difference in saccadic amplitude was statistically significant according to the Mann–Whitney test (U = −75.63, *p* < 0.001).

The difference in the amplitude distributions of saccades performed during convergence and divergence movements was also statistically significant (U = 4.28, *p* < 0.001), although the differences in the medians (IQR) of the two distributions were subtle (1.10 deg (1.98 deg) for saccades done during convergence, and 1.02 deg (1.72 deg) for saccades done during divergence) (Figure 3D,E).

A more detailed analysis of medians (IQR) of the saccadic amplitude distributions is presented in Table 2, where amplitudes are reported as a function of the fusional vergence sign (positive and negative) and direction of the vergence movement (convergence and divergence). The tendency towards greater saccadic amplitudes during the experience of diplopia reported above was true regardless of the direction of the vergence movement and the sign of the fusional vergence tested. Moreover, during fusion, saccades done during the convergence period of the NFV measurement had greater amplitude than those during the divergence period. The opposite trend was observed for the saccades done during the PFV measurement. The differences between all pairwise comparisons were statistically significant (*p* ≤ 0.007). During diplopia, similar trends were found, as both the Kruskal–Wallis and the pairwise comparison tests showed significant differences in the medians between each pair (*p* < 0.001). Table 2 also contains the number of saccades that occurred during fusion and diplopia. The total number of saccades exhibited during the experience of diplopia during the PFV test was considerably smaller than that during the NFV measure. For the former test, there was a strong significant correlation between the number of saccades that occurred during diplopia and the time participants were experiencing diplopia (ρ = 0.96, *p* < 0.001), indicating that those participants who made more saccades during diplopia also spent more time in this condition. As expected, participants experienced diplopia for longer during the measurement of NFV. However, in this case, this amount of time did not correlate significantly with the number of saccades exhibited (ρ = 0.27, *p* = 0.111).

**Figure 4:**
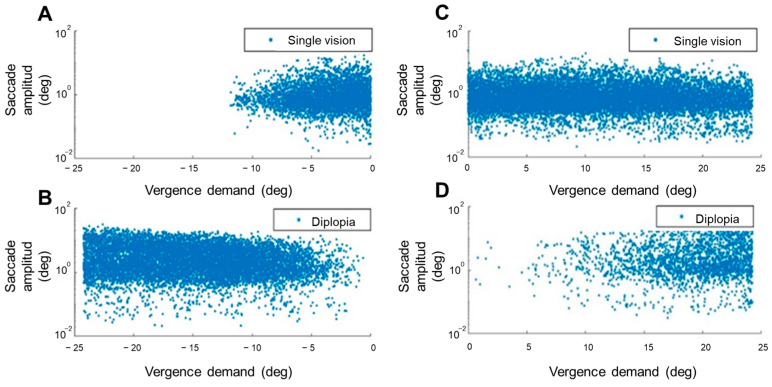
Saccadic amplitude as a function of vergence demand. Amplitude of saccades done during the negative fusional vergence (**A**,**B**) and positive fusional vergence measures (**C**,**D**) represented as a function of the vergence demand and the experience of single vision (**A**,**C**) or diplopia (**B**,**D**).

**Figure 5:**
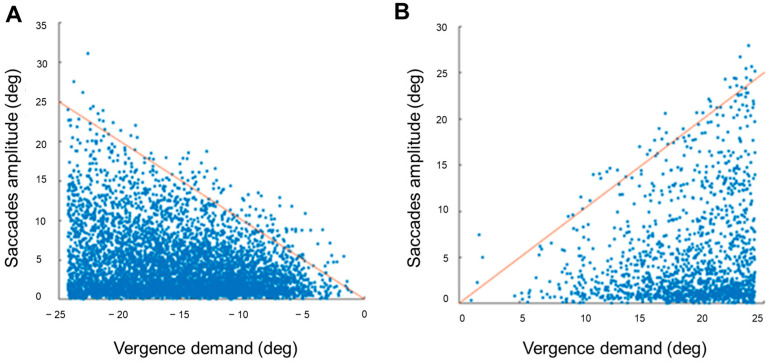
Amplitude of horizontal saccades done during the experience of diplopia as a function of vergence demand. (**A**) represents the saccades done during the negative fusional vergence measure, and (**B**) represents the saccades done during the positive fusional vergence measure. The red line corresponds to the identity line.

Saccadic amplitude was analyzed as a function of vergence demand (Figure 4 and Figure 5). A moderate significant correlation was found between the vergence demand and the amplitude of saccades that occurred during the NFV measure (ρ = −0.35, *p* < 0.001). A weaker significant correlation was found for the saccades done during the PFV measure (ρ = 0.11, *p* < 0.001). The sign of the correlations indicated larger saccadic amplitudes with increasing vergence demand. A more in-depth analysis showed that these correlations were stronger considering only the saccades done during the experience of diplopia (ρ = −0.15, *p* < 0.001 and ρ = 0.11, *p* < 0.001 for negative and positive fusional vergence measures, respectively) than for saccades done during single vision (ρ = 0.07, *p* < 0.001 and ρ = −0.02, *p* = 0.005) (Figure 4). These results indicated correlations between vergence demand and saccadic eye movement amplitudes, with varying strengths depending on the sign of fusional vergence test (positive or negative) and whether participants experienced single or double vision. In actuality, the stronger correlations found considering saccades done during the experience of diplopia might reflect the presence of not only small involuntary saccades, but also larger saccades to alternate fixation between the two diplopic images whose relative distance increases with vergence demand. Figure 5 represents the amplitude of these saccades.

### 3.3. Direction

**Figure 6:**
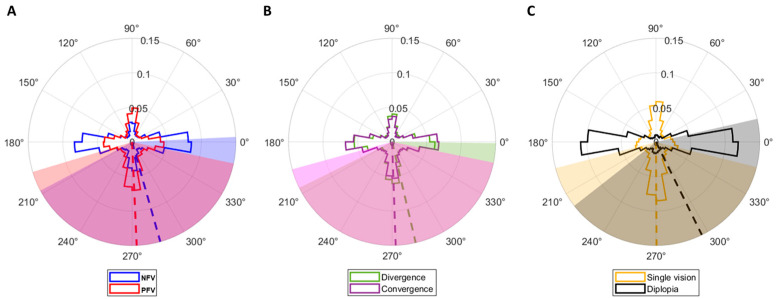
Polar histograms representing the saccades’ direction distributions in different conditions. (**A**) shows the direction distributions of saccades that occurred during the measurements of negative, in blue, and positive, in red, fusional vergence amplitudes. (**B**) shows the saccade’s direction distributions as a function of the direction of the underlying vergence movement, in green, divergence, and, in purple, convergence. In the negative fusional vergence test, first the test stimulated divergence movements from 0 PD to 45 PD, and then convergence from 45 PD to 0 PD. In the positive fusional vergence test, the sequence of movements was the opposite. First, the stimuli drove convergence movements from 0 PD to 45 PD, and then divergence from 45 PD to 0 PD. (**C**) represents the saccade’s direction distributions depending on whether they occurred during periods of single vision, in orange, or diplopia, in black. For all plots, the dashed lines represent the means, while the standard deviations are shaded in pastel colors, and the overlapping region indicates directions shared by both conditions.

The distribution of saccades’ direction is shown in Figure 6. Vertical saccades (±22.5 deg from 90 deg) were more prevalent (39%) than horizontal (±22.5 deg from 0 deg) ones (31%) during the measurement of PFV amplitudes, whereas the opposite trend was observed during the measurement of NFV amplitudes (52% horizontal and 22% vertical) (Figure 6A). The means ± SD direction of saccades done during the positive and negative fusional vergence measures were 285.78 ± 77.80 deg and 272.55 ± 75.74 deg, respectively. The Watson–Wheeler test showed statistically significant differences between the two distributions (*p* < 0.001). However, this behavior was rather idiosyncratic, as only 17 participants (half of the total sample) exhibited significant differences between the distributions of direction of saccades done during positive and negative fusional vergence measures. This difference cannot be explained by the direction of the underlying vergence movement, as saccades that occurred during divergence and convergence movements showed similar direction distributions with mean ± SD directions of 283.17 ± 76.85 deg and 272.00 ± 76.62 deg, respectively (*p* = 0.099, Watson–Wheeler test) (Figure 6B). In this case, only two participants exhibited significant differences when considered individually. Instead, the experience of diplopia or single vision made a difference in the direction of saccades. During fusion, there were more vertical saccades (46.41%) than horizontal (22.68%). In the contrary, during the experience of diplopia, most saccades were horizontal (68.28%) (Figure 6C). This trend was true regardless of whether the saccades occurred during the positive or negative fusional vergence amplitudes measure. The mean ± SD direction of saccades done during the experience of single vision and diplopia was 270.30 ± 75.60 deg and 296.23 ± 78.16 deg. The Watson–Wheeler test showed statistically significant differences between the two distributions (*p* < 0.001), and all except seven participants exhibited the same behavior. This marked difference in direction distributions together with the correlation of saccadic amplitude with vergence demand, especially for saccades done during diplopia, might reflect the saccades done to read the column of letters used as stimulus during single vision and the saccades done to alternate fixation between the two images during diplopia.

### 3.4. Direction Conjugacy

**Figure 7:**
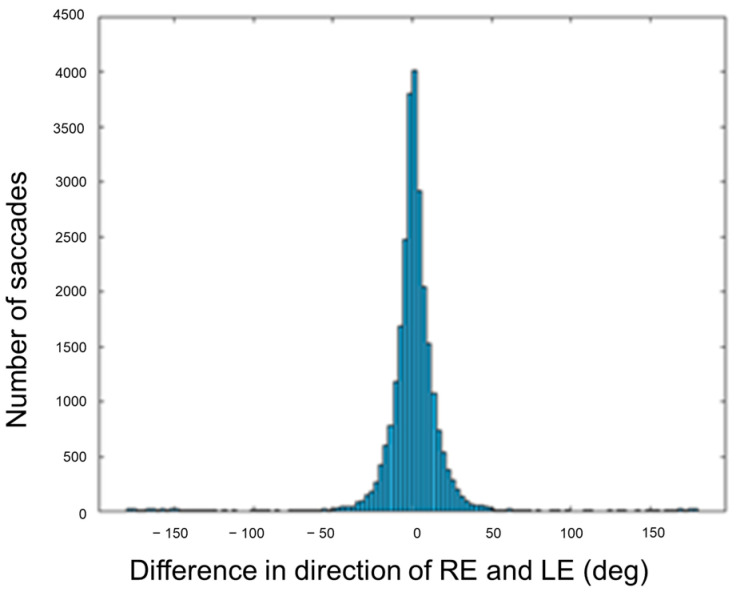
Histogram of the difference in saccade direction of the right eye (RE) and left eye (LE). A difference of 0 deg means that the two eyes moved in the same direction, whereas a difference of ±180 deg means that the saccades had opposite direction in the two eyes.

The mean ± SD of the difference in saccade direction between the right and left eyes was 0.61 ± 29.53 deg (Figure 7). In addition, direction conjugacy of saccades was also analyzed, decomposing their amplitude in the two eyes into their signed vertical and horizontal components. In this sense, those saccades whose vertical or horizontal components had opposite signs in the two eyes were considered disconjugate saccades.

**Figure 8:**
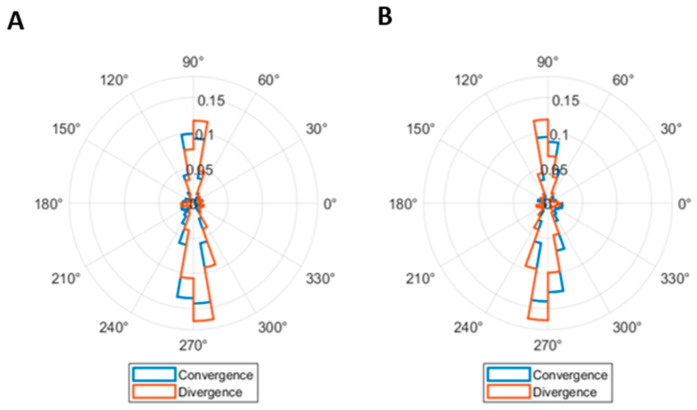
Direction of the right (**A**) and left (**B**) eyes during horizontally disconjugate saccades. The distributions of direction of saccades done during convergence (blue) and divergence (orange) are similar.

The total number of disconjugate saccades was 6062. The purpose of this analysis was to determine whether disconjugate saccades contributed to the concurrent vergence movement. Therefore, only the horizontally disconjugate saccades were considered. There were 3589 horizontally disconjugate saccades. During the NFV test, a total of 1224 saccades were observed, with 576 occurring during convergence, and 648 during divergence. Of the horizontally disconjugate saccades during the divergence movement, 67.9% had a divergent component and could be regarded as corrective saccades, while 43.1% of the horizontally disconjugate saccades done during the convergence movement had a convergent component. In contrast, more horizontally disconjugate saccades were done during the PFV test, with a total of 2365 saccades. Approximately half of them (1170) occurred during convergence, and the other half (1195) during divergence. Among these horizontally disconjugate saccades during the convergence movement, 59.8% exhibited a convergent component, and 58.2% during divergence had a divergent component, thus qualifying as corrective saccades. In summary, there was no clear trend of most horizontally disconjugate saccades having convergent or divergent components in the same direction as the stimulus, thus contributing to reducing the vergence error. This effect is also illustrated in Figure 8, where the direction of the right and left eyes during horizontally disconjugate saccades is represented.

## 4. Discussion

The present study aimed to investigate the characteristics of saccades that occurred during the positive and negative fusional vergence test. The analyzed variables included the frequency, main sequence, saccadic amplitude, direction, and conjugacy between eyes.

These results suggest that the characteristics of saccades showed a marked difference in performance depending on the direction of the underlying vergence movement [48], and agree that the oculomotor system adapts differently when faced with vergence challenges [49,50]. These facts are related to previous studies that found that convergence and divergence movements are controlled by different neural control systems [51,52], a finding with potential clinical implications for diagnosing and treating binocular vision disorders.

The number of saccades increased in each repetition of the PFV test but remained stable during the NFV test, which might reflect some level of fatigue after being exposed to high convergence demands [53,54]. On the other hand, the measurement of PFV is less repeatable than the measure of NFV [13], which could be associated with increased variability in oculomotor performance across repetitions. Other authors have explained this higher variability by the neural coupling between vergence and accommodation [3,55].

Another finding of the present study is that the differences in the saccades’ amplitude and direction distributions during the measurement of positive and negative fusional vergence amplitudes might be explained by the periods of diplopia. The average break point for the NFV was 13.62 Δ, whereas that for the PFV was 37.37 Δ. The recovery points were 10.26 Δ and 27.70 Δ for the negative and positive fusional vergence, respectively. This means that, as expected, the periods of diplopia were longer during NFV measurements because it is a more demanding task, whereas fusion was maintained more easily during PFV measurements. Another related aspect to consider is that, as the fixation stimulus was a vertical optotype, it is feasible that participants read the letters while changing the vergence angle, resulting in a high prevalence of small vertical saccades. In contrast, when fusion was no longer maintained and participants experienced diplopia, they perceived two separate columns of letters. As a result, they could make larger horizontal saccades to alternate fixation between the two diplopic images. The saccades’ direction distributions represented in Figure 6C provide strong evidence of this behavior. In addition, the higher percentage of larger saccades during the NFV measure and the scaling of the amplitude of horizontal saccades that occurred during diplopia according to the vergence demand shown in Figure 5 provide additional evidence of the presence of saccades alternating between the two diplopic images.

During the measurement of NFV amplitudes, a larger number of saccades was exhibited during diplopia compared to the PFV amplitudes test (Table 2). In the latter test, a strong correlation implied that those participants who exhibited more saccades during diplopia also experienced this condition for a longer period of time. However, the same trend was not observed in the measurement of NFV, suggesting the influence of other factors beyond time in explaining differences in number of saccades. A possible explanation might be that the ability to recover fusion is more challenging during NFV than during PFV testing, thus requiring the recruitment of more saccades to facilitate vergence movements [55]. Previous studies on saccade–vergence interactions have demonstrated a facilitating role of saccades under certain vergence conditions [22,50,55,56]. In this context, individuals with oculomotor dysfunctions may present impaired vergence control and reduced fusional vergence amplitudes. Interestingly, individuals with dyslexia have been shown to exhibit larger amplitudes of saccades and weaker and less stable vergence responses than controls [57,58].

Moreover, dyslexic children with altered saccadic movements often display reduced attentional capacities and binocular vision anomalies, which could be linked to atypical vergence dynamics [59]. Several studies have also reported a decreased number of vergence responses containing saccades in patients with convergence insufficiency after a successful vision therapy protocol [32,60]. Although the present study focused on participants with normal binocular vision, the characterization of saccadic behavior during highly demanding vergence tasks has direct clinical relevance, as numerous ocular and neurological conditions [61,62], including strabismus [63], amblyopia [64], dyslexia [65,66], or Parkinson’s disease [67], among others, are associated with abnormal eye movement control. Establishing normative values for saccade–vergence interaction metrics in healthy individuals may, therefore, aid clinicians in improving the diagnosis and monitoring of different conditions and contribute to the development of more targeted and effective interventions, ultimately enhancing patient outcomes.

The direction of the two eyes during most saccades was similar (Figure 7), consistent with the definition of saccades as conjugate eye movements [32,68]. However, saccades performed during vergence movements may not always be perfectly conjugated, as suggested by some authors [69,70] and observed in a small percentage of saccades in the current study. Horizontally disconjugate saccades during vergence movements might play a corrective role in reducing vergence error, as found in previous studies [21], and analogously to catch-up saccades during smooth pursuit eye movements [71]. However, our results did not support this hypothesis, as the horizontal component of disconjugate saccades, in general, was not in the correct direction to reduce vergence errors (Figure 8).

A limitation of eye tracking research is the replicability of the results. Several studies have found differences in fusional vergence amplitudes depending on the method used to stimulate vergence [13,14,18]. Thus, the haploscopic setup used in the current study might lead to different fusional vergence amplitudes than the Risley prisms or prism bars commonly used in clinics. However, the findings of the effects of vergence demand on saccadic characteristics should be reproducible if an eye tracker with similar specifications to the EyeLink 1000 Plus and similar algorithms to detect saccades are used. Eye tracking technology in optometric clinical testing offers numerous advantages, including detailed objective data on eye movements, despite certain limitations such as high costs, complex procedures that require specific training, and difficulties in data interpretation. Despite these disadvantages, eye tracking technology is a valuable tool for the diagnosis of binocular vision disorders. Another limitation of this study is that differences in saccadic behavior between myopic and hyperopic individuals were not analyzed [72], and the potential influence of refractive error was not considered. These factors can be addressed in future research.

Future work using eye tracking technology may not only enhance our understanding of normal visual processing and oculomotor performance but also open new avenues for investigating the interaction between saccades and vergence in a range of clinical disorders. Moreover, additional research could focus on investigating the potential of saccades and microsaccades as objective markers for predicting the vergence break and recovery points of single binocular vision.

## 5. Conclusions

The present results contribute to the understanding of how the visual system adapts saccades in response to different disparity vergence demands during the measurement of fusional vergence amplitudes in adults with typical binocular vision. Saccadic amplitude, direction, and frequency varied as a function of the underlying vergence movement and the experience of diplopia or single vision. Furthermore, the normative values established in this study may support future research into the saccade–vergence interaction in populations with binocular vision disorders, with the potential to improve diagnostic accuracy and to inform the development of more targeted treatment approaches.

## Figures and Tables

**Table 1:** Descriptive statistics for the number of saccades done in each repetition of the NFV and PFV measure.

Test		Median (Number of Saccades)	IQR (Number of Saccades)
Negative fusional vergence	Repetition 1	127.00	53.00
	Repetition 2	122.50	58.75
	Repetition 3	126.00	37.75
Positive fusional vergence	Repetition 1	139.00	70.50
	Repetition 2	141.50	59.50
	Repetition 3	149.50	51.50

**Table 2:** Descriptive statistics for the saccades’ amplitude distribution as a function of the fusional vergence sign (positive and negative) and direction of the vergence movement (convergence divergence).

	Negative Fusional Vergence		Positive Fusional Vergence	
	Divergence	Convergence	Convergence	Divergence
For all saccades				
Number of saccades	6028	7075	6968	7413
Median (deg)	1.26	1.89	0.71	0.88
IQR (deg)	2.38	3.37	0.89	1.32
For saccades during fusion				
Number of saccades	2146	1798	6165	6049
Median (deg)	0.72	0.95	0.66	0.75
IQR (deg)	0.90	1.28	0.78	0.96
For saccades during diplopia				
Number of saccades	3882	5277	803	1364
Median (deg)	1.26	2.43	2.31	1.79
IQR (deg)	2.32	3.81	4.71	3.72

## Data Availability

Data will be shared upon request.

## Abbreviations

The following abbreviations are used in this manuscript:

NFV	Negative fusional vergence
PFV	Positive fusional vergence
SD	Standard deviation
CISS	Convergence Insufficiency Symptom Survey
Δ	Prism diopters
cpm	Cycles per minute
deg	Degrees
